# Multidisciplinary collaborative treatment of 48 cases of village cluster acute nitrite poisoning: A case series study

**DOI:** 10.1097/MD.0000000000045959

**Published:** 2025-11-21

**Authors:** Yanmin Huang, Xuebing Tang, Tao Zhou, Hailin Ruan

**Affiliations:** a Department of Emergency Medicine, The Fourth Hospital affiliated to Guangxi Medical University/Liuzhou Worker's Hospital, Liuzhou, Guangxi, China; b Department of Medical Administration, People Hospital of Rongshui Miao Autonomous County, Liuzhou, Guangxi, China; c Department of Emergency Medicine, Liuzhou Railway Central Hospital/Guangxi Medical University Affiliated Liuzhou railway Central Hospital, Liuzhou, Guangxi, China.

**Keywords:** mass poisoning, methylene blue, multi-department collaboration, nitrite poisoning, primary hospital

## Abstract

**Rationale:**

This report aims to evaluate the effectiveness of a multidepartment collaborative response system in managing a large-scale nitrite poisoning incident in rural China, focusing on organizational strategies and clinical interventions.

**Patient concerns:**

Forty-eight villagers collectively developed symptoms after a village banquet, primarily including dizziness, vomiting, and cyanosis of the lips and nail beds. The onset of symptoms occurred 0.5 to 1.5 hours after ingestion.

**Diagnoses:**

Based on the diagnostic criteria for acute dietary nitrite poisoning and confirmed by Centers for Disease Control and Prevention (CDC) laboratory tests showing elevated nitrite levels in biological samples (>15 mg/kg), all patients were definitively diagnosed with acute nitrite poisoning.

**Interventions:**

A multidepartment collaborative emergency response mechanism was activated, implementing prehospital transport and graded management: Mild cases (37 patients) received induced vomiting, oxygen therapy, and intravenous methylene blue (1–2 mg/kg). Severe cases (10 patients) received intensified methylene blue therapy alongside organ function support, with eligible patients undergoing continuous renal replacement therapy.

**Outcomes:**

All 47 hospitalized patients recovered and were discharged after treatment. One patient died before reaching the hospital due to underlying diseases, deeper intoxication, and delayed medical attention.

**Lessons:**

This rescue effort validates the effectiveness of the multidepartment collaborative model for responding to mass poisoning incidents in rural areas. Key lessons include: Graded transport and early methylene blue administration are crucial for improving prognosis. Cross-institutional information exchange enhances response efficiency. There is an urgent need to strengthen primary-level emergency response capacity and public health education. This framework provides a replicable model for similar resource-limited settings.

## 1. Introduction

On January 30, 2024, a mass acute nitrite poisoning incident involving 48 cases occurred in a village in Wangdong Township, Rongshui County, Liuzhou City, Guangxi Zhuang Autonomous Region, triggered by a housewarming banquet. The event featured sudden onset and a large number of affected individuals, posing severe challenges to local medical rescue capabilities.

Nitrite poisoning is a critical emergency. Its strong oxidizing property converts ferrous iron in hemoglobin to ferric iron, inducing methemoglobinemia, tissue oxygenation impairment, and vasodilation.^[1,2]^ The high water solubility of nitrite contributes to rapid symptom manifestation,^[3]^ including cyanosis, hypoxia, altered consciousness, arrhythmia, and death.^[4]^ Toxicological studies indicate that a single intake exceeding 0.2 g can cause symptoms in adults, and approximately 3.0g can be lethal.^[5]^ Treatment must be initiated immediately upon diagnosis. Crucially, the early administration of the specific antidote methylene blue is key to improving rescue success rates,^[6]^ alongside supportive therapies such as emesis induction, gastric lavage, catharsis, and intravenous fluids.

The epidemiology of nitrite poisoning exhibits regional variations. In the United States, intentional nitrite ingestion has become a recognized suicide method.^[7]^ Furthermore, several countries, including Canada, have reported rising numbers of intentional sodium nitrite ingestions and associated fatalities.^[8]^ In contrast, accidental poisoning is more common in China,^[9]^ often resulting from consuming spoiled pickled vegetables, improperly stored cooked dishes, or meats cured with excessive nitrite.^[10]^ Notably, nitrite’s physical resemblance to table salt, frequently compounded by unclear labeling, leads to accidental ingestion,^[11]^ often presenting as mass poisoning events.^[12]^

Mass casualty incidents (involving ≥3 individuals) are characterized by acute onset, rapid progression, and large numbers of affected people.^[13]^ Untimely intervention significantly increases the risk of severe illness and death. This risk is particularly pronounced in rural villages, where residents often lack awareness of nitrite poisoning hazards, potentially leading to delayed medical attention or limited resources contributing to fatalities. This incident underscores the challenges in preventing and controlling mass food poisoning in rural areas, highlighting the urgent need to strengthen the management of industrial salt and food safety education.

This study retrospectively analyzes the treatment process and outcomes of this event to explore the application value of a multi-department collaborative emergency system in responding to such mass casualty incidents, aiming to provide a reference for the prevention, control, and treatment of mass poisoning events in rural villages.

## 2. Case report

### 2.1. Epidemiological background and exposure source

This case report details an acute mass food poisoning event involving 48 villagers (26 males, 22 females; age range 19–69 years, mean 49 ± 12.11 years) from a village in Wangdong Township. All were definitively diagnosed with nitrite poisoning. The cause was identified as the chef at a banquet mistakenly using industrial salt instead of edible salt during cooking, contaminating the dishes consumed. This study received approval from the Ethics Committee of Liuzhou Worker's Hospital. Case data were retrospectively obtained from electronic medical records, and granted a waiver of informed consent by the Ethics Committee.

### 2.2. Clinical manifestations and prehospital assessment

The celebratory banquet commenced at 7:30 am in a buffet style. Symptoms developed collectively 0.5 to 1.5 hours after eating (see Table 1). Based on diagnostic criteria for acute dietary nitrite poisoning (see Table 2), 37 patients were classified as mild cases, 10 as severe cases, and 1 died.

**Table 1:** Symptoms or signs of nitrite poisoning.

	Symptom or sign	Number of cases (n)	Incidence (%)
Hypoxia symptoms	Cyanosis	43	90
Nervous system	Dizziness/headache	46	96
–	Generalized weakness	33	69
Digestive system	Nausea/vomiting	26	54
Respiratory system	Dyspnea	11	23
Circulatory system	Palpitations	11	23
–	Shock	1	2

**Table 2:** Severity classification of poisoning.

Severity grade	Diagnostic criteria	Number of cases (n)	Proportion (%)
Mild	Neurological and/or digestive symptoms only	37	77
Severe	Accompanied by respiratory and/or circulatory dysfunction	10	21
Death	Prehospital cardiac and respiratory arrest	1	2

### 2.3. Multi-department collaborative response mechanism

**Figure 1.**
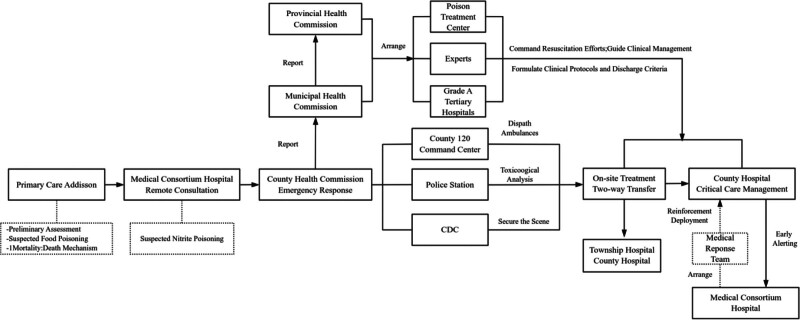
Multi-tiered multi-department response framework for mass nitrite poisoning management.

The Township Hospital received the initial cohort of suspected food poisoning cases. Remote consultation with Medical Consortium Hospital suggested mass nitrite poisoning, triggering immediate escalation to the County Health Commission. The County 120 Command Center(the regional emergency medical services coordination hub) dispatched multi-jurisdictional ambulances for coordinated patient transport. Simultaneously, the County Hospital activated its mass poisoning protocol, mobilizing medical consortium resources. The Municipal/Provincial Health Commissions deployed toxicology experts from tertiary Grade-A hospitals and prefecture-level toxicology emergency centers to command resuscitation, guide evidence-based management and Establish treatment protocols with discharge criteria. Concurrently, CDC laboratory analysis confirmed diagnostic nitrite levels in biological samples (>15 mg/kg), while Public Security secured the incident site. (see Fig. 1 for integrated workflow.)

### 2.4. On-site treatment and transport management

The Township Hospital received several patients presenting with dizziness, vomiting, and cyanosis of lips/nail beds at 10:00 am based on history and clinical presentation, mass food poisoning was suspected, and treatments including induced vomiting, fluid infusion, and oxygen therapy were administered. Subsequent remote consultation with upper-level hospitals suggested nitrite poisoning. An ambulance arrived at the village at 10:35 am; assessment revealed 1 deceased patient (not transported), while the remaining patients were transported in batches according to severity level.

The County 120 Command Center, notified around 10:40 am, mobilized ambulance resources. Guided by expert teams, they coordinated the bi-directional handover and transport of patients between the township health center and county hospital ambulances for treatment at the county hospital. Concurrently, emergency medications, including methylene blue, were gathered from county and city reserves. The county hospital received its first patient en route at 12:00 pm Key response time points are detailed in Table 3.

**Table 3:** Key time points in mass poisoning incident response.

Event	Time	Time elapsed since initial medical contact (min)
Onset of first symptoms	08:00–09:00	–
Initial assessment at Township Hospital	10:00	0
Ambulance arrival at the scene	10:35	35
County 120 command Center dispatch for transfer	10:40	40
First patient handover during transfer	12:00	120
CDC laboratory confirmation of diagnosis	13:00	180

CDC = the Centers for Disease Control and Prevention.

Continuous cardiac monitoring, dynamic assessment, oxygen therapy, and intravenous access were maintained throughout transport. Multi-departmental communication ensured timely access to toxicology results; some patients received intravenous methylene blue at the township health center or during transport. Due to the remote location, rugged roads, and approximately 2-hour transport time, many patients experienced tension and anxiety; medical staff provided psychological support.

### 2.5. In-hospital treatment system

The hospital emergency command group (comprising the deputy director, Medical Affairs Department, Nursing Department, Emergency Department, etc) coordinated the rescue: Digestive department beds were vacated; emergency teams were formed; the entire Emergency Department was put on standby; and the intensive care unit prepared for potential admissions. The Medical Affairs Department coordinated personnel, supplies, and information management, assigning dedicated staff for patient registration, data summarization, and reporting. The Pharmacy Department ensured adequate medication supplies, and the Logistics Department allocated equipment such as gastric lavage machines.

Upon arrival of the 47 patients, rapid triage was performed again: 37 mild cases and 10 severe cases. Patients were diverted based on severity via a green channel for emergency cases (treatment first, formalities later), with mild cases admitted to the Digestive Department and severe cases to the intensive care unit. Treatment protocol included (see Table 4): antidote: methylene blue (1–2 mg/kg) administered intravenously in 25% glucose solution, repeated if necessary, combined with intravenous vitamin C. Routine management: oxygen therapy, induced vomiting, gastric lavage, catharsis, ensuring airway patency, preventing aspiration, and maintaining warmth. Supportive treatment: anti-shock measures, etc. Blood purification: continuous renal replacement therapy was performed for critically ill patients meeting the indications. Monitoring: observation of skin/mucosa and turgor, vital signs, recording 24-hour fluid intake/output, repeated methemoglobin level measurements, and prevention of complications.

**Table 4:** Key in-hospital treatment measures for nitrite poisoning.

Treatment Measure	Number of patients (n)	Dosage/frequency
Methylene blue IV push	47	1–2 mg/kg (repeated ≤3 times prn)
Vitamin C IV infusion	47	3 g/d
CRRT	1	48 h

CRRT = continuous renal replacement therapy.

### 2.6. Clinical outcomes

Among the 48 nitrite poisoning patients, 47 recovered and were discharged following treatment involving multi-sectoral collaboration led by the Health Department, encompassing expert teams, hospital alliance partners, CDC, Public Security, and hospital departments. One patient died prehospital. The cause of death was the interaction of underlying diseases (alcoholic cirrhosis, chronic obstructive pulmonary disease, hypertension), deeper intoxication due to higher intake, lack of awareness regarding nitrite poisoning, and failure to seek medical attention, leading to a fatal interplay of chronic illness, acute poisoning, and medical delay.

## 3. Discussion

### 3.1. The core role of methylene blue and graded management practice

Methylene blue is the specific antidote, with its effects being dose-dependent. At low doses (1–2 mg/kg), it is converted to the reduced form via glucose-6-phosphate dehydrogenase, facilitating the reduction of methemoglobin to normal hemoglobin by donating hydrogen ions, thereby restoring oxygen-carrying capacity; the reduced methylene blue can be reoxidized and function cyclically.^[14]^ Conversely, higher concentrations of methylene blue (e.g., 7 mg/kg) can paradoxically reduce splanchnic perfusion,^[15]^ necessitating strict dosage control. Vitamin C inhibits endogenous nitrite synthesis and scavenges free radicals. Continuous renal replacement therapy is an effective modality for treating severe poisoning,^[16]^ efficiently removing toxins and inflammatory mediators, stabilizing organ function, preventing complications, and improving prognosis.

In this case series, a graded treatment strategy was applied to all 47 treated patients. Mild cases resolved rapidly with induced vomiting, oxygen therapy, and 1 to 2 mg/kg methylene blue. Severe cases received intensified methylene blue therapy and organ support, ultimately achieving a 100% in-hospital survival rate. This outcome underscores the pivotal role of methylene blue and the value of graded management in mass poisoning incidents.

### 3.2. Value of multi-department collaborative response

The incidence and mortality of mass nitrite poisoning show an increasing trend.^[17]^ Establishing a public health emergency response system is crucial for enhancing the capacity to handle such incidents.^[18]^ This event occurred in a remote village approximately 100 km from the county town, highlighting the triple pressures faced in resource-limited settings: geographic isolation (causing transport delays), medical scarcity (inadequate treatment capacity), and mass casualty overload. Under these circumstances, the multi-department collaborative emergency system, through vertical integration (e.g., cloud-based medical consortium consultations enabling provincial expert decision-making at the frontline) and horizontal linkage (e.g., CDC toxicology testing coordination), transformed fragmented resources into an efficient “survival chain.” This systematically compressed the “exposure-to-antidote time window,” laying the foundation for successful treatment.

This case series demonstrates that multi-department collaboration is an effective solution when confronting the triple challenge of resource scarcity, limited capacity, and geographic barriers. It provides a replicable technical paradigm for resource-scarce regions globally – particularly applicable to emergency scenarios involving transport bottlenecks, delayed toxicology confirmation, and primary-level manpower shortages.

### 3.3. Weaknesses in primary-level medical emergency systems and areas for improvement

This mass nitrite poisoning event in a village revealed 3 core weaknesses. The primary weakness is the severe inadequacy of independent treatment capacity in township hospitals, where the number and professional competence of medical staff are insufficient to manage mass casualty incidents, necessitating rapid external reinforcement. Secondly, there is a critical gap or shortage of essential antidotes (such as methylene blue) at village and township levels, creating a fracture in the emergency resource chain. Thirdly, information silos exist across county, township, and village levels, leading to delayed patient data and decision-making. It is recommended to strengthen the emergency response capabilities of primary healthcare personnel, establish professional health emergency teams, increase funding guarantees, and ensure that primary institutions conduct health education, emergency drills, and maintain material reserves.

### 3.4. Preventive strategies for exposure source control

One patient in this series, unaware of the poisoning’s danger, did not seek treatment and died. Enhanced public education is recommended, utilizing diverse channels such as door-to-door campaigns, school education, and social media.^[19]^ Content should cover food preservation, container labeling, and self-rescue measures after accidental ingestion. Governments and relevant agencies should regularly organize health knowledge lectures and emergency drills, encouraging villager participation to enhance their emergency awareness and practical skills.^[20]^

## 4. Conclusion

This case study demonstrates that implementing a multi-department collaborative response mechanism and a graded management strategy may contribute to improved treatment outcomes for poisoning incidents in remote areas. Strengthening primary-level emergency response capacity and public education are urgently needed. System optimization should focus on: upgrading collaboration mechanisms; Building primary-level emergency response capacity; targeted public education. The effectiveness of these interventions requires validation through multi-center studies. Subsequent multi-center controlled studies are recommended to provide more reliable evidence-based guidance for the standardized management of mass poisoning incidents.

Supplemental Digital Contents (“Patient Medical Record, WST86-1996”) are available for this article (https://links.lww.com/MD/Q666, https://links.lww.com/MD/Q667).

## Author contributions

**Investigation:** Tao Zhou.

**Resources:** Xuebing Tang.

**Writing – original draft:** Yanmin Huang.

**Writing – review & editing:** Hailin Ruan.

## Supplementary Material




