# Supplementary material for: Multidisciplinary collaborative treatment of 48 cases of village cluster acute nitrite poisoning: A case series study
Source: Medicine (Baltimore). 2025 Nov 21;104(47):e45959. doi: 10.1097/MD.0000000000045959 (PMC12643662; doi:10.1097/MD.0000000000045959)
Supplement: Supplementary file 2 [file medi-104-e45959-s002.pdf]

中华人民共和国卫生行业标准

食源性急性亚硝酸盐中毒诊断标准及处理原则

WS/T 86—1996

Diagnostic criteria and principles of management of dietary acute nitrite poisoning

1 主题内容与适用范围

本标准规定了食源性急性亚硝酸盐中毒的诊断标准、判定原则及处理原则。

本标准适用于因食用含亚硝酸盐超量食物或混有亚硝酸盐食物而引起的急性中毒。

2 引用标准

GB/T 5009.33—1996 食品中亚硝酸盐与硝酸盐的测定方法

GB 8788 职业性急性苯的氨基、硝基化合物(三硝基甲苯除外)中毒诊断标准及处理原则

GB 14938—94 食物中毒诊断标准及技术处理总则

3 诊断标准

3.1 流行病学特点

进食了腐烂变质的蔬菜,腌制不久的咸菜或存放过久的熟菜,使用过量的亚硝酸盐腌肉,或误将亚硝酸盐当作食盐烹调的食物。

3.2 临床表现

食源性急性亚硝酸盐中毒是进食了含有较大量的亚硝酸盐食物后,在短期内引起的以高铁血红蛋白症为主的全身性疾病。

3.3 临床诊断

轻者有头晕、头痛、乏力、胸闷、恶心、呕吐,口唇、耳廓、指(趾)甲轻度紫绀等,高铁血红蛋白在10%～30%。重者可有心悸、呼吸困难,甚至心律紊乱、惊厥、休克、昏迷、皮肤、粘膜明显紫绀,高铁血红蛋白往往超过50%。

3.4 实验室诊断

3.4.1 剩余食物、呕吐物或胃内容物作亚硝酸盐测定(按GB/T 5009.33),含量超标。

3.4.2 血液高铁血红蛋白测定(按GB 8788附录A),含量超过10%。

4 判定原则

4.1 符合流行病学调查的特点,确认中毒由亚硝酸盐引起。

4.2 临床表现符合亚硝酸盐中毒。

4.3 剩余食物或呕吐物中检出超过限量的亚硝酸盐。

4.4 血液中高铁血红蛋白含量超过10%。

中华人民共和国卫生部1997-01-11批准　　1997-09-01实施

5 处理原则

5.1 按 GB 14938 执行。

5.2 特效解毒药：高铁血红蛋白症可用美兰，大量维生素 C 也可应用。

附加说明：

本标准由卫生部卫生监督司提出。

本标准由卫生部食品卫生监督检验所、中国预防医学科学院劳动卫生与职业病研究所负责起草。

本标准主要起草人张临夏、鲁锡荣。

本标准由卫生部委托技术归口单位卫生部食品卫生监督检验所负责解释。
